# HPV16 infection decreases vaccine-induced HPV16 antibody avidity: the CVT trial

**DOI:** 10.1038/s41541-022-00431-x

**Published:** 2022-03-29

**Authors:** Sabrina H. Tsang, John T. Schiller, Carolina Porras, Troy J. Kemp, Rolando Herrero, John Schussler, Monica S. Sierra, Bernal Cortes, Allan Hildesheim, Douglas R. Lowy, Ana Cecilia Rodríguez, Byron Romero, Nicolas Çuburu, Jaimie Z. Shing, Ligia A. Pinto, Joshua N. Sampson, Aimée R. Kreimer, on behalf of the Costa Rica HPV Vaccine Trial Group, Bernal Cortés, Paula González, Rolando Herrero, Silvia E. Jiménez, Carolina Porras, Ana Cecilia Rodríguez, Allan Hildesheim, Aimée R. Kreimer, Douglas R. Lowy, Mark Schiffman, John T. Schiller, Mark Sherman, Sholom Wacholder, Ligia A. Pinto, Troy J. Kemp, Mary K. Sidawy, Wim Quint, Leen-Jan van Doorn, Linda Struijk, Joel M. Palefsky, Teresa M. Darragh, Mark H. Stoler

**Affiliations:** 1 National Cancer Institute, National Institutes of Health, Bethesda, MD USA; 2 Agencia Costarricense de Investigaciones Biomédicas (ACIB), formerly Proyecto Epidemiológico Guanacaste, Fundación INCIENSA, San José, Costa Rica; 3 HPV Immunology Laboratory, Leidos Biomedical Research, Inc., Frederick National Laboratory for Cancer Research, Frederick, MD USA; 4 Early Detection and Prevention Section, International Agency for Research on Cancer, World Health Organization, Lyon, France; 5 Information Management Services, Silver Spring, MD USA; 6 Independent Consultant, San José, Costa Rica; 7 Westat, Rockville, MD USA; 8 Agencia Costarricense de Investigaciones Biomédicas (ACIB), formerly Proyecto Epidemiológico Guanacaste, PEG, Fundación INCIENSA, San José, Costa Rica; 9 Independent Consultant, San José, Costa Rica; 10 Mayo Clinic, Jacksonville, FL USA; 11 Medstar Georgetown University Hospital, Washington, DC USA; 12 DDL Diagnostic Laboratory, Rijswijk, Netherlands; 13 University of California, San Francisco, CA USA; 14 Emeritus, University of Virginia, Charlottesville, VA USA

**Keywords:** Cancer epidemiology, Adjuvants, Infection

## Abstract

The HPV vaccine has shown sustained efficacy and consistent stabilization of antibody levels, even after a single dose. We defined the HPV16-VLP antibody avidity patterns over 11 years among women who received one- or three doses of the bivalent HPV vaccine in the Costa Rica HPV Vaccine Trial. Absolute HPV16 avidity was lower in women who received one compared to three doses, although the patterns were similar (increased in years 2 and 3 and remained stable over the remaining 8 years). HPV16 avidity among women who were HPV16-seropositive women at HPV vaccination, a marker of natural immune response to HPV16 infection, was significantly lower than those of HPV16-seronegative women, a difference that was more pronounced among one-dose recipients. No differences in HPV16 avidity were observed by HPV18 serostatus at vaccination, confirming the specificity of the findings. Importantly, point estimates for vaccine efficacy against incident, six-month persistent HPV16 infections was similar between women who were HPV16 seronegative and seropositive at the time of initial HPV vaccination for both one-dose and three-dose participants. It is therefore likely that this lower avidity level is still sufficient to enable antibody-mediated protection. It is encouraging for long-term HPV-vaccine protection that HPV16 antibody avidity was maintained for over a decade, even after a single dose.

## Introduction

Antibody avidity is a measure of the collective binding strength (affinities) of a polyclonal antibody response to a defined antigen^1^. As a measure of the functional maturation of the humoral immune response^2^, avidity depends on the intrinsic affinity of the antibody for the antigen, the valency of the antibody/antigen interaction (monovalent vs. bivalent binding in the case of IgG), and the structural arrangement of the interacting components. Increases in antibody avidity after vaccination with protein antigens is often observed, the result of somatic hypermutation in the variable regions of immunoglobin genes, which normally occurs in lymph node germinal centers (GCs). However, there is a paucity of information on the changes in the avidity of the polyclonal antibodies induced by vaccines over the course of many years or the influence of booster doses or immune responses from preexisting infection on the strength and durability of the avidity.

Clinical trials of the HPV virus-like particle (VLP) vaccines such as the Costa Rica HPV Vaccine Trial (CVT) provide an opportunity to address these questions, because it has demonstrated that three, two, or even a single dose of the bivalent HPV vaccine (Cervarix^TM^) result in durable antibody levels against the vaccine-targeted HPV types; this is correlated with durable protection against infection by these types and as well as several cross-protected types^3^. The most recent data from CVT have shown that this degree of protection is sustained for over a decade following initial vaccination^4,5^. Specifically, there is evidence demonstrating that the anti-HPV16 or 18 antibody levels did not decline between years 4 and 11 and were significantly higher than those induced by natural infection, regardless of the number of vaccine doses received, although one-dose titers continued to be significantly lower than two- and three-dose titers^4^.

Previous publications from CVT have evaluated antibody avidity^3,6,7^. In women who received three doses of the bivalent HPV vaccine, anti-HPV16-VLP antibody avidity steadily increased between years 1 and 4, as measured in a chaotrope-based enzyme-linked immunosorbent assay (ELISA)^6^. At Year 4, a small difference in HPV16 antibody avidity between women who received one or three doses of the HPV vaccine was observed, which remained stable for both dose groups at Year 7^3^. These findings suggested that booster vaccine doses provide only a small increase in long-term anti-HPV16 antibody avidity.

It is of considerable interest to measure the evolution of affinity during the initial years after single-dose vaccination and to further define the long-term durability of the avidity response by dose group. With more than a decade of active follow-up of HPV-vaccinated women in our trial, we assessed HPV16 antibody avidity out to 11 years post-vaccination among women who received the recommended three-dose HPV vaccine regimen and compared that to women who received only a single dose. In addition, we examined the effect of previous or current HPV16 infection on HPV16 antibody avidity to the vaccine’s VLP antigens after vaccination, since the HPV vaccines are widely administered to sexually active young women. Our study aimed to increase our understanding of the antibody response to the HPV vaccine, shed light on how a single dose of the bivalent HPV vaccine provides durable protection against HPV infection, and provide predictions on how future virus-like display vaccines may perform.

## Results

### Characteristics of study participants at enrollment

We demonstrated representativeness of our sample selection and comparability in characteristics between one-dose and three-dose groups at study enrollment and thus first HPV vaccination with respect to age, HPV16 serostatus and HPV18 serostatus, and HPV DNA status (HPV16/18 positive, any HPV positive, or HPV negative) (Supplementary Table 1). The number of follow-up visits attended, and the number of visits tested for avidity, were also similar between dose groups. Among those included in the current analysis, 71% in the one-dose and 75% in three-dose groups were HPV16 seronegative at first HPV vaccination, while 74% in the one-dose group and 76% in the three-dose group were HPV18 seronegative at first HPV vaccination. More than one-half of the women were HPV16/18 DNA negative at first HPV vaccination: 51% and 65% in the one-dose and three-dose groups, respectively.

### Antibody avidity comparing one-dose and three-dose participants

**Fig. 1:**
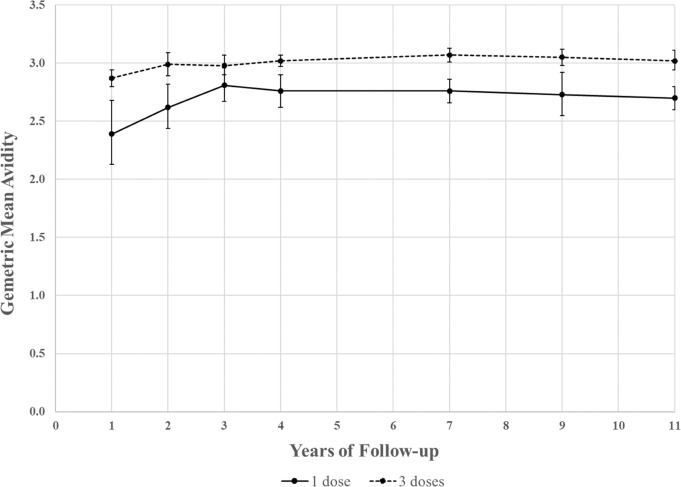
Antibody avidity over time among participants who were HPV16 seronegative at first HPV vaccination. Dots represent the geometric mean antibody avidity index for the one-dose group (solid line) and the three-dose group (dashed line), and the error bars represent 95% confidence intervals (CI). Regression lines are estimated by regressing log(avidity) on either an intercept, an indicator for the second study visit, and/or study year, and reporting on the parameter the parameter 100(eβ-1).

For one-dose recipients (198 women with 747 serum samples), Geometric Mean of the Avidity Index (GMA) ranged from 2.39 to 2.81 for years 1 through 11 (Table 1, Fig. 1). The greatest increase in avidity was observed in the early years (9.8% increase in Year 2 and 7.2% increase in Year 3). Avidity remained stable in subsequent years: the GMA in Year 4 was 2.76 and 2.70 in Year 11; the *p*-for-trend for between years 4 and 11 was 0.28 (Table 1).

**Table 1:** Antibody avidity over time among one-dose and three-dose participants who were HPV16 seronegative at first HPV vaccination.

		One-dose (*N* = 747 samples)			Three-dose (*N* = 1115 samples)				One-dose/Three-dose
Year of follow-up	*n*	GMA (95% CI)	IQR	% change^†^	*n*	GMA (95% CI)	IQR	% change^†^	Ratio (95% CI)
Year 1	52	2.39 (2.13–2.68)	2.25–3.07	N/A	161	2.87 (2.80–2.94)	2.68–3.16	N/A	0.83 (0.74–0.94)
Year 2	56	2.62 (2.44–2.82)	2.34–3.10	9.8	142	2.99 (2.89–3.09)	2.82–3.31	4.0	0.88 (0.81–0.95)
Year 3	51	2.81 (2.67–2.96)	2.60–3.11	7.2	119	2.98 (2.90–3.07)	2.84–3.31	−0.2	0.94 (0.89–1.00)
Year 4	123	2.76 (2.62–2.90)	2.65–3.13	−1.9	191	3.02 (2.97–3.07)	2.87–3.26	1.2	0.91 (0.87–0.96)
Year 7	162	2.76 (2.66–2.86)	2.54–3.15	0.1	225	3.07 (3.01–3.13)	2.94–3.40	1.8	0.90 (0.86–0.94)
Year 9	125	2.73 (2.55–2.92)	2.58–3.16	−1.1	136	3.05 (2.98–3.12)	2.96–3.32	−0.8	0.90 (0.83–0.96)
Year 11	178	2.70 (2.60–2.80)	2.46–3.08	−1.2	141	3.02 (2.94–3.11)	2.89–3.32	−0.8	0.89 (0.85–0.93)
*p*-for-trend*		0.28				0.85			

*N* total number of samples, *n* number of samples tested per study visit, *GMA* Geometric Mean of the Avidity Index, *IQR* interquartile range, *CI* confidence intervals, *N/A* not applicable.

^†^% change was calculated based on two consecutive visits.

*p-for-trend within dose group, over time in years 4–11.

For three-dose recipients (321 women with 1115 serum samples), GMA ranged from 2.87 to 3.07 during years 1 through 11. The greatest increase in avidity was 4.0% in Year 2. Avidity remained stable during years 4 through 11, measuring 3.02 both in years 4 and 11; the *p*-for-trend between years 4 and 11 was 0.85. Overall, the antibody avidity of three-dose recipients was higher than that of one-dose recipients throughout the study period, although the difference was small for years 3 through 11. On average, the GMA ratio between the one-dose and three-dose groups was 0.90 (95% CI: 0.86–0.93); at the peak of the avidity response, in Year 3, the ratio was 0.94 (0.89–1.00) (Table 1).

In addition to the GMAs being stable over time in both the one-dose and three-dose participants, the avidities for individual participants similarly tended to be stable over time (Supplementary methods, Supplementary Figs. 1–5; Supplementary Table 2).

### Antibody avidity comparing participants who at first HPV vaccination were HPV seropositive vs. HPV seronegative

**Fig. 2:**
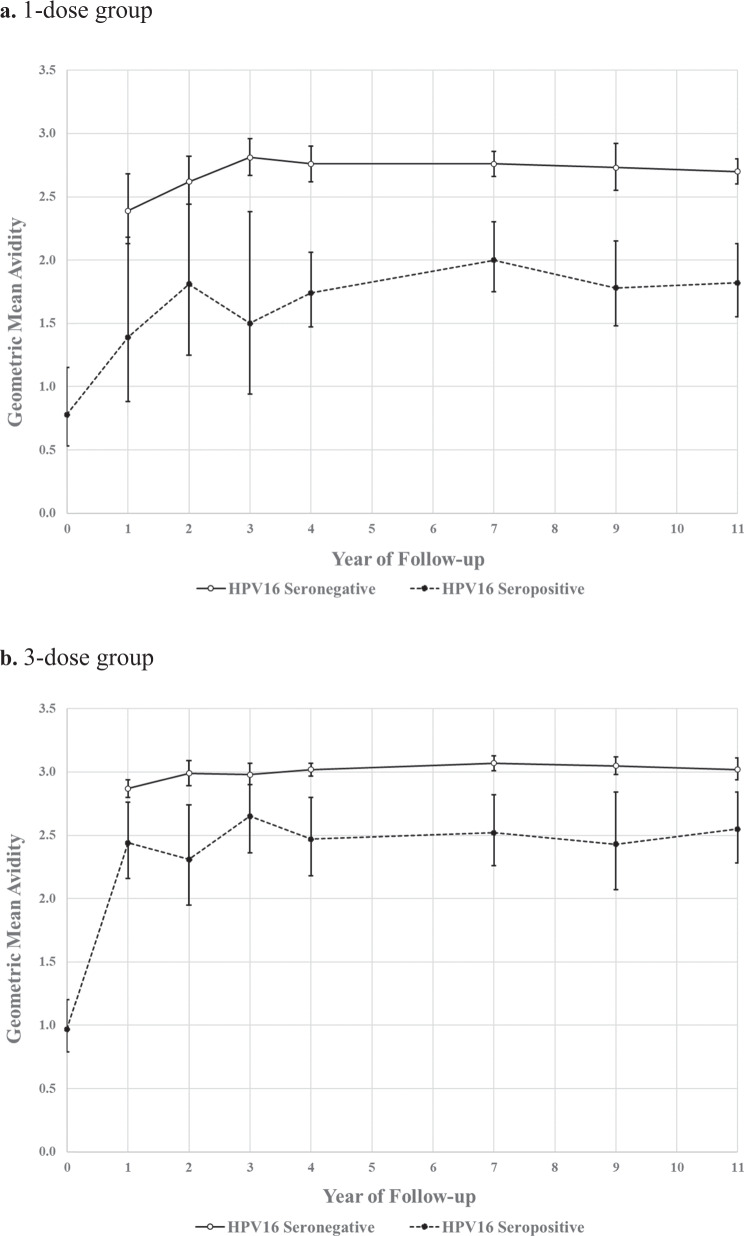
HPV16 antibody avidity over time, stratified by number of HPV vaccine doses received and HPV16 serostatus at time of initial HPV vaccination. HPV16-seronegative status at first HPV vaccination is represented by the solid line and HPV16 seropositive at first HPV vaccination is represented by the dashed line. Dots represent the geometric mean antibody avidity index for the one-dose group (**a**) and the three-dose group (**b**) and the error bars represent 95% confidence intervals (CI). Regression lines are estimated by regressing log(avidity) on either an intercept, an indicator for the second study visit, and/or study year, and reporting on the parameter the parameter 100(eβ-1).

For women who were HPV16 seropositive at first HPV vaccination, which reflected current and/or prior infection, it was possible to measure the avidity of these antibodies. In serum collected just prior to vaccination (27 samples in the one-dose and 43 samples in the three-dose group), the GMAs in baseline seropositive subjects were 0.78 (0.53–1.15) and 0.97 (0.79–1.20) in one-dose and three-dose recipients, respectively, which are substantially lower compared to post-vaccination (Table 2). Vaccination of this group resulted in a substantial increase in avidity. Unexpectedly, however, their avidity level remained significantly lower than that of women who were HPV16-seronegative at the time of first vaccination; this difference was particularly pronounced among the one-dose recipients (Fig. 2 and Table 2). Over the nested study follow-up period, 198 women in the one-dose group had 774 study visits (540 HPV16-seronegative samples and 234 HPV16-seropositive samples) and 321 women in the three-dose group had 1158 study visits (853 HPV16-seronegative samples and 305 HPV16-seropositive samples). The average GMA ratios between baseline seropositive and seronegative women were 0.66 (0.57–0.77) in the one-dose group and 0.82 (0.75–0.90) in the three-dose group, a significant difference (*p* = 0.017). These ratios remained relatively constant throughout the study period (Table 2).

**Table 2:** Antibody avidity over time among one-dose and three-dose participants, stratified by HPV16 serostatus at first HPV vaccination.

Study visit	One dose (*N* = 774)							Three dose (*N* = 1158)						
	HPV16 seronegative			HPV16 seropositive			Seropositive/Seronegative	HPV16 seronegative			HPV16 seropositive			Seropositive/Seronegative
	*n*	GMA (95% CI)	% change†	*n*	GMA (95% CI)	% change†	Ratio (95% CI)	*n*	GMA (95% CI)	% change^†^	*n*	GMA (95% CI)	% change^†^	Ratio (95% CI)
First HPV vaccination	0	N/A	N/A	27	0.78 (0.53–1.15)	N/A	N/A	0	N/A	N/A	43	0.97 (0.79–1.20)	N/A	N/A
Year 1	42	2.39 (2.13–2.68)	N/A	10	1.39 (0.88–2.18)	77.0	0.58 (0.36–0.93)	117	2.87 (2.80–2.94)	N/A	44	2.44 (2.16–2.76)	150.4	0.85 (0.75–0.96)
Year 2	47	2.62 (2.44–2.82)	9.8	9	1.81 (1.25–2.62)	30.5	0.69 (0.47–1.00)	109	2.99 (2.89–3.09)	4.0	33	2.31 (1.95–2.74)	−5.2	0.77 (0.65–0.92)
Year 3	44	2.81 (2.67–2.96)	7.2	7	1.50 (0.94–2.38)	−17.2	0.53 (0.33–0.85)	93	2.98 (2.90–3.07)	−0.2	26	2.65 (2.36–2.98)	14.5	0.89 (0.79–1.00)
Year 4	81	2.76 (2.62–2.90)	−1.9	42	1.74 (1.47–2.06)	16.3	0.63 (0.53–0.75)	152	3.02 (2.97–3.07)	1.2	39	2.47 (2.18–2.80)	−6.9	0.82 (0.72–0.93)
Year 7	119	2.76 (2.66–2.86)	0.1	43	2.00 (1.75–2.30)	15.2	0.73 (0.63–0.84)	168	3.07 (3.01–3.13)	1.8	57	2.52 (2.26–2.82)	2.2	0.82 (0.73–0.92)
Year 9	82	2.73 (2.55–2.92)	−1.1	43	1.78 (1.48–2.15)	−10.9	0.65 (0.54–0.80)	106	3.05 (2.98–3.12)	−0.8	30	2.43 (2.07–2.84)	−3.7	0.80 (0.68–0.93)
Year 11	125	2.70 (2.60–2.80)	−1.2	53	1.82 (1.55–2.13)	1.9	0.67 (0.57–0.79)	108	3.02 (2.94–3.11)	−0.8	33	2.55 (2.28–2.84)	5.0	0.84 (0.75–0.94)
Overall*	**540**	**2.70 (2.61–2.80)**		**234**	**1.80 (1.55–2.08)**		**0.66 (0.57–0.77)**	**853**	**3.01 (2.96–3.05)**		**305**	**2.48 (2.27–2.71)**		**0.82 (0.75–0.90)**
*p*-for-trend**		0.28			0.79				0.85			0.82		

*N* total number of samples, *n* number of samples tested per study visit, *GMA* Geometric Mean of the Avidity Index, *CI* confidence intervals, *N/A* not applicable.

^†^% change was calculated based on two consecutive visits.

*Overall GMAs calculated over years 1–11. *P* value comparing overall GMA ratios was 0.017, presented in bold.

** *p*-for-trend within dose group, over time in years 4–11.

**Fig. 3:**
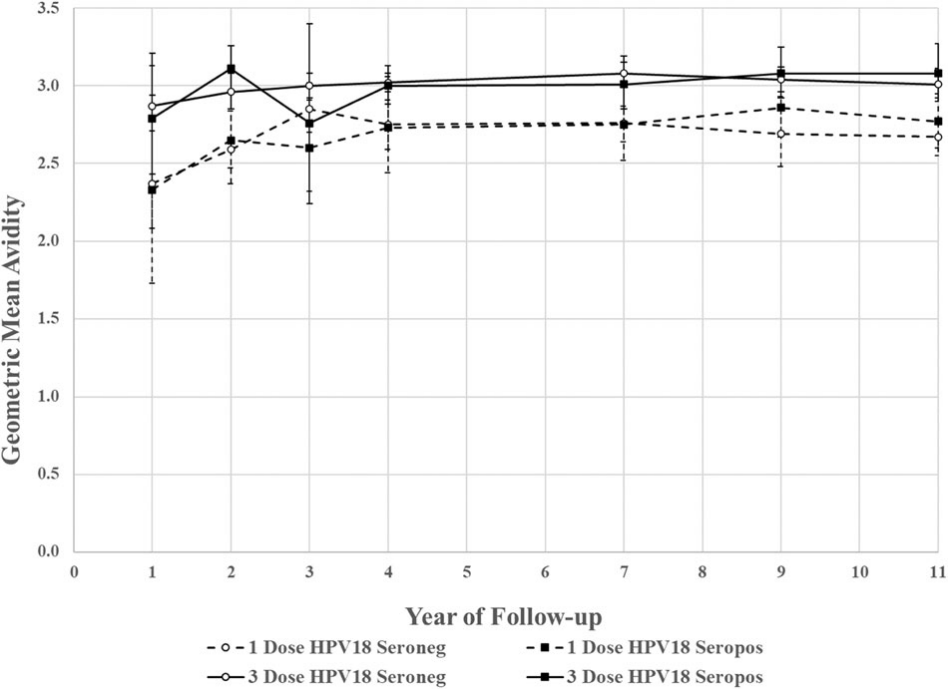
HPV16 antibody avidity over time among HPV16 seronegative at initial HPV vaccination, stratified by number of HPV vaccine doses received and HPV18 serostatus at time of initial HPV vaccination. The solid lines indicate the three-dose group, stratified by HPV18 seronegative (empty circles) and HPV18 seropositive (squares) at first HPV vaccination. The dashed lines indicate the one-dose group, stratified by HPV18 seronegative (empty circles) and HPV18 seropositive (squares) at first HPV vaccination. Error bars represent 95% confidence intervals (CI). Regression lines are estimated by regressing log(avidity) on either an intercept, an indicator for the second study visit, and/or study year, and reporting on the parameter the parameter 100(eβ-1).

To determine whether seropositivity at first HPV vaccination to another HPV type might also be associated with lower post-vaccination HPV16 avidity, we compared the avidity for the HPV16-seronegative women who were HPV18 seropositive prior to HPV vaccination with those who were seronegative for both HPV16 and HPV18 prior to HPV vaccination. In contrast to the women who were HPV16 seropositive prior to HPV vaccination, no differences in HPV16 avidity were observed between HPV18 seropositives and seronegatives prior to HPV vaccination among the three- or one-dose groups (Fig. 3). Each of the HPV18 seropositive: seronegative ratios was approximately 1.0 and all confidence intervals (CIs) included 1.0, indicating no significant difference.

### Dose-stratified vaccine efficacy against incident persistent HPV16 infections, stratified by HPV16 serological status at time of vaccination

Among three-dose participants, similarly high vaccine efficacy against incident persistent HPV16 infection was observed after four years of follow-up among women who were HPV16 seronegative (95.4%; 95% CI 88.5–98.6%) and HPV16 seropositive (93.4%; 95% CI 62.8–99.7%) at the time of initial HPV vaccination. In the smaller one-dose group, vaccine efficacy was 100% among both the HPV16 seronegative (95% CI 60.1–100.0%) and HPV16 seropositive (95% CI −171% to 100.0%) women at the time of initial HPV vaccination, although, statistical power for seropositive women was limited due to small sample size (Table 3).

**Table 3:** Bivalent HPV16 vaccine efficacy against incident, 6-month persistent HPV16 infections among women who were HPV DNA negative through year 2, stratified by HPV16 serological status at time of vaccination and dose.

	Vaccine		Control		Vaccine efficacy
Group	*n*/*N*	Attack rate per 1000 women	*n*/*N*	Attack rate per 1000 women	% (95% CI)
HPV16 DNA negative and HPV16 seronegative					
One-dose	0/115	0.0 (0.0–25.7)	8/101	79.2 (37.5–144.8)	100.0% (60.1–100.0%)
Three-dose	4/1965	2.0 (0.6–4.9)	84/1898	44.3 (35.7–54.2)	95.4 (88.5–98.6%)
HPV16 DNA negative and HPV16 seropositive					
One-dose	0/41	0.0 (0.0–70.5)	2/32	62.5 (10.6–191.5)	100.0% (−171.0 to 100.0%)
Three-dose	1/582	1.7 (0.1–8.4)	15/580	25.9 (15.1–41.4)	93.4% (62.8–99.7%)

*CI* confidence interval.

### Correlation between antibody avidity and antibody level at first HPV vaccination

To evaluate the possibility of confounding by differences in the amount of VLP-specific antibodies in specific sera, we evaluated correlations between antibody avidity and concentration, two variables independently measured by laboratory assays. Among women who were HPV-seronegative at first HPV vaccination, the time-averaged Pearson correlation coefficient between the log-avidity index and log-antibody level was 0.04 (95% CI −0.10 to −0.18) for the one-dose group and 0.14 (0.01–0.27) for the three-dose group (Supplementary Table 3, Supplementary Fig. 4). The correlations ranged from -0.13 (Year 11) to 0.27 (Year 1) for one-dose recipients, and from 0.11 (Year 2) to 0.37 (Year 11) for three-dose recipients. While one-dose recipients generally showed a lack of correlation between antibody avidity and antibody level, a slight but consistent positive correlation was observed in three-dose recipients. A similar pattern was observed among women who were HPV seropositive at first HPV vaccination (Supplementary Table 3, Supplementary Fig. 5).

## Discussion

The current study has made several observations about the development and maintenance of antibody avidity during the 11 years following bivalent HPV vaccination, including comparisons between one dose and three doses and the woman’s serostatus at the time of HPV vaccination. First, we determined that avidity after a single vaccine dose continues to mature over a period of several years with absolute values only slightly lower than that of three doses. Second, the high-level post-vaccination avidity persists through year 11 after one or three doses, which parallels the durability we have previously observed for antibody levels^4^. Third, we unexpectedly found that the post-vaccination avidity in women who were HPV16 seropositive at first HPV vaccination increased after vaccination but remained lower than the avidity women who were HPV16 seronegative at first HPV vaccination. These results contrast with observations that plateau-phase HPV16 antibody titers were independent of HPV16 serostatus at time of first HPV vaccination^8^. Fourth, despite lower avidity among HPV16-seropositive women compared to HPV16-seronegative women, one-dose and three-dose vaccine efficacy against incident, 6-month persistent HPV16 infections was similarly robust regardless of serological status at initial vaccination. Fifth, we did not observe a close correlation between serum antibody levels and avidity, implying they may be largely non-overlapping parameters. This strengthens the interpretation of the findings, suggesting observations in this work are specific to antibody avidity and not driven by antibody concentration.

There have been other investigations into HPV vaccine-induced avidity, albeit with different findings, likely due to differences in either study or laboratory methodology. Sankaranarayanan et al.^9^, reported no difference in HPV6, 11, 16, or 18 L1 antibody avidity at a single time point, 18 months post-vaccination, in girls receiving one, two, or three doses. Several differences in study design could account for the difference in results, including differences in the vaccines (Gardasil^®^ vs Cervarix^TM^), the subjects were younger (aged 10–18 years), the use of a different chaotrop in the ELISA (urea vs guanidine hydrochloride), and the avidity measurements were determined for a single concentration of chaotrop rather over a range of concentrations as in our study, which might permit detection of subtler differences. Pasmans et al.^10^ also reported no difference in antibody avidity to HPV16 L1 at 5 years post-vaccination in girls aged 12–16 years who received one, two, or three doses of Cervarix^TM^ an average of five years earlier. However, avidities were also determined for single concentration of a different chaotrope (ammonium thiocynate). Perhaps more importantly, the ages of the one and three-dose Cervarix^TM^ recipients in Pasmans’ work differed, 12 and 16 years respectively, whereas the vaccinees in our study were the same ages. This could influence the avidity results in two ways. Antibody responses to HPV VLPs are in general superior in children prior to puberty. It is reasonable to suspect that this difference may extend to avidities. In addition, Pasmans et al. did not stratify by HPV16 serostatus at baseline. It is reasonable to postulate that there are more HPV16 seropositives in the 16-year-old three-dose group than in the 12-year-old one-dose group. Our finds document that avidities tend to be lower in seropositives at baseline, so differential seropositivity in the three-dose group would differentially lower their mean affinity, the net result that they could appear to be the same as one-dose recipients.

The avidity that developed from one dose continued to increase over the first three years after vaccination, which is a year longer than it took to reach maximal avidity after three doses. The average levels of avidity during years 3 through 11 remained 5–10% lower after one dose compared with three doses, a smaller difference than the differences in antibody levels after one versus three doses^4^. At the individual level, avidities within each participant similarly tended to be stable over time. As only a small minority of the women were likely exposed to HPV16 virus during the trial^4^, the ongoing high avidity is attributable to the durability of the vaccine response.

There are at least two possible explanations for the continued affinity maturation over several years after only a priming dose. One could be that there is prolonged retention of the vaccine antigen in the GCs. Although complete denaturation or degradation of a typical protein antigen might be expected over a shorter timeframe, HPV VLPs are exceptionally stable structures, and their polyvalency might promote their extended retention on follicular dendritic cells^11^. Alternatively, the gradual increase in avidity might be attributable to the preferential survival during this period of plasmablasts that received the strongest signaling through their B cell receptors^12^, i.e., had the highest affinity for the VLPs, after which any survivors would have become established, long-lived plasma cells (LLPCs). Regardless of the mechanism, the sustained high avidity levels of HPV16 antibodies through year 11 make it likely that once the peak level of avidity has been reached for a given woman, her LLPCs continue to produce consistent antibody levels whose avidity is also consistent.

Women who were HPV16 seropositive at vaccination had lower avidities in follow-up: the GMA ratio for the HPV16-seropositive women vs. the HPV16-seronegative women was significantly lower (0.66) for one dose than for the three doses (0.82). The lower HPV16 avidity levels appear to be antigen-specific, rather than being attributable to a more general immune perturbation induced by mucosal HPV infection, as the HPV16 avidity for women who were HPV18 seropositive at first HPV vaccination was similar to the HPV18 seronegative women. Therefore, it is likely that the prior low-level mucosal exposure of a specific antigen substantially influences the subsequent quality of the antibody response upon parenteral re-exposure to what is essentially the same antigen. The HPV18 results also serve to distinguish the response to HPV vaccination from the concept of “original antigenic sin” where, as in the case of influenza infection, prior exposure to one virus strain prevents efficient induction of antibodies to unique epitopes from a distinct, but antigenically related, strain^13^.

The mechanism responsible for the reduced antibody avidity after prior mucosal exposure is uncertain. One possible explanation is that low-avidity memory B cells (mBCs), preferentially generated after mucosal infection, can be driven into LLPCs after secondary exposure to antigen by parenteral vaccination, but remain “imprinted” with a generally lower avidity B cell receptor, despite presumably having participated in a secondary GC reaction and therefore a second round of somatic hypermutation. A previous study found that most of the HPV16-VLP monoclonal antibodies (MoAb) generated from infection-induced mBCs were non-neutralizing, while most of the MoAb generated after vaccination were strongly neutralizing^14^. This observation suggests that the antibodies resulting from infection may generally be of lower avidity, despite the fact that HPV16-VLP-specific IgG genes from mBCs induced by infection and vaccination had similar numbers of somatic mutations^14^ A second possibility is that the avidity levels measured after vaccination of HPV-exposed women reflect the sum of the lower avidities of the antibodies produced by LLPCs generated from naive B cells after infection and those of higher avidities generated after vaccination. A subset of women do appear to generate LLPCs after infection, as indicated by the persistence of serum antibodies after the apparent clearance of infection^15^. Mechanistically, this might appear to be the simplest explanation. However, the GMTs after natural infection are 10-fold lower than those seen at the plateau phase after single-dose vaccination and 40-fold lower than that after three doses. It is difficult to reconcile the apparently small contribution of infection-induced antibodies to the total antibody levels and the substantial effect on the avidities, especially for the three-dose recipients.

The hypothesis that LLPCs after boosting are mostly generated from naive B cells, rather than lower avidity mBCs, is supported by several observations. First, the plateau levels of antibodies maintained after VLP vaccination are almost additive by dose. We repeatedly observe that, in the plateau phase, HPV16-VLP antibody levels after three doses are only about fourfold higher than after one dose. Second, the quality of the antibodies after priming and booster doses is similar, as measured here by avidity and previously by the ratio of neutralizing to binding titers^4,16^. Therefore, one explanation for our observations is that the induction of LLPCs largely follows the same pathway initiated by an interaction with naive B cells after priming and boosting. Consistent with this hypothesis, the recruitment of cognate mBCs into secondary GCs was limited in mouse models of vaccination and influenza infection. Rather most of B cells recruited into the secondary GCs appeared to be antigen inexperienced, likely naive, B cells. Since it is widely held that B cells much go through a GC reactive to evolve into LLPCs, these findings imply that most of the durable antibody responses after booster immunization would be derived from naive B cells^17^.

It is important to emphasize that, although vaccination of women who were HPV16 seropositive at first HPV vaccination had lower avidities than the seronegative women, this did not translate into suboptimal protection against HPV infection. Specifically for three-dose participants, strong and significant vaccine efficacy was observed against incident, 6-month persistent HPV16 infections after 4 years of follow-up among women who were HPV16 seropositive at first HPV vaccination (VE of 93%), which was similar to that among HPV16-seronegative women (VE of 95%). One-dose vaccine efficacy was also similar by HPV16 serostatus (both VE of 100%); however, results for HPV16-seropositive women were underpowered resulting in broad CIs. These results corroborate our understanding of HPV vaccine protection and durability. First, one-dose HPV vaccination provided to young adult women, some of whom might be expected to have been HPV exposed prior to vaccination, continues to provide strong protection against HPV infection^4^. Second, one vaccine dose induced a substantial increase in avidity compared to the avidity measured at first HPV vaccination after natural infection. These results indicate that, in addition to quantity, the quality of the antibody response to vaccination remains far superior to that induced by infection. The lower avidity levels that develop after infection may partially explain why the antibody-mediated protection induced by infection appears to be less consistent than protection induced by vaccination, even in vaccinees with low antibody levels after vaccination, e.g., cross-type reactive ones^18^.

In some situations, a close correlation has been observed between avidity/affinity of vaccine-induced antibodies and their in vitro neutralizing activities, as in the case of an MF59 adjuvated pandemic influenza vaccine^19^. However, we did not observe such a correlation here between avidity and antibody titer, as measured our ELISA assay, which has been shown to closely correlate with neutralizing activity^16^. Instead, the results from an experimental vesicular stomatitis (VSV) model may be more relevant to the HPV vaccine. In the VSV model, increasing avidity contributed to in vivo protection only until a threshold was reached, after which protection was dependent on higher antibody levels but not on higher avidity^20^. If this model is relevant to the HPV vaccine, even the lower post-vaccination avidity levels that develop in the women who were HPV seropositive at first HPV vaccination are high enough to enable protection.

Given the exceptionally high vaccine efficacy observed for even a single dose of the HPV vaccine^4^, there is no evidence that prior HPV infection compromises the protection afforded by the HPV vaccines against subsequent exposure. Yet, our findings raise the possibility that suboptimal preexisting immunity generated by other viral mucosal infections might have similarly deleterious effects on the long-term antibody repertoire after subsequent vaccination, which could be relevant for vaccines where the avidity threshold for protection may be higher than for HPV. For example, might asymptomatic SARS-2 infection limited to the upper respiratory mucosa induce low-avidity antibodies that could result in lower vaccine-induced antibody avidity compared with immunologically naive vaccinees? Given our results, it may be prudent to investigate this possibility.

## Methods

### Study design of CVT

Between 2004 and 2005, 7466 women 18–25 years of age in Costa Rica were enrolled and randomized to receive either the AS04-adjuvanted HPV16/18 vaccine (Cervarix^TM^; GlaxoSmithKline Biologicals, Rixensart, Belgium) or a control hepatitis A vaccine (GlaxoSmithKline Biologicals) in a 1:1 ratio at 0, 1, and 6 months, and were followed for four years in CVT^21^ (NCT00128661). At enrollment and annual follow-up visits, participants provided a serum sample, and for sexually experienced women, a pelvic exam was performed at which cervical cells were collected for cytology and HPV DNA testing^21^.

At the end of the blinded phase, participants in the HPV-vaccinated arm were invited to stay in the CVT observational study^22^ and followed biennially in years 7, 9, and 11, when each clinic visit consisted of a pelvic exam with collection of a cervical sample and a serum sample, for virologic and immunologic endpoint assessments, respectively.

Approximately 20% of women in the CVT received fewer than three doses of their assigned vaccine, even though all women were randomized to receive three doses^23^. Reasons for missing vaccine doses were independent of trial arm and largely involuntary, with major reasons being pregnancy and colposcopic referral^23^. In the HPV vaccine arm, this resulted in 275 women receiving a single dose of the HPV vaccine and 2964 women receiving the standard three-dose regimen.

### Human subjects

All study protocols were approved by the U.S. National Cancer Institute (NCI) Institutional Review Boards and the corresponding Costa Rican Institutional Review Board; all participants signed written informed consent.

### Sample selection for antibody avidity testing

For this study, we focused on the enrollment and follow-up study visits at Years 0, 1, 2, 3, 4, 7, 9, and 11. All eight timepoints were included to fully describe the long-term kinetics of antibody avidity over time. Avidity at Year 0 (pre-vaccination; enrollment visit) was only evaluated in women who were HPV16 seropositive at first HPV vaccination, providing insight into the avidity of natural infection-induced antibody and the effect of prior infection on avidity responses to the vaccine. Our selection was based on availability of prior IgG ELISA results for HPV16 (tested over the course of multiple CVT studies^3,4,16,24–27^ at the Frederick National Laboratory for Cancer Research, Frederick, Maryland), necessary to control for the concentration of antibody added to the avidity assay, and further restricted to those who contributed two or more serum samples over the course of 11 years (excluding the enrollment visit). After applying these inclusion criteria, our selection yielded 198 one-dose women (with 747 follow-up samples) and 321 three-dose women (with 1115 follow-up samples). We did not evaluate responses to two doses of the vaccine, because most two-dose women in CVT received their second dose after one month, which is an inferior regimen that is not considered to be of public health relevance.

### Laboratory methods for avidity testing

The assessment of anti-HPV16-VLP antibody IgG avidity was performed as described previously^7,28^. Briefly, microtiter plates were coated with HPV16 L1 VLPs, and each serum sample was assayed at a single dilution, ranging from 1/100 to 1/120,000 for all samples evaluated, which yielded an absorbance reading of 1.0 ± 0.5 as previously determined in an HPV16 ELISA. Guanidine-HCl (GuHCl) was added to the samples at various concentrations (0.5–3.5 M) to elute low-avidity antibodies. The concentration of GuHCl that reduced the optical density by 50% compared to sample wells without GuHCl treatment defined the Avidity Index, which serves as the quantitative readout of antibody avidity, specifically measuring the strength of the binding under increasingly stringent binding conditions. There was a narrow range for antibody avidity results and in some cases (*N* = 134) the assay results were beyond the upper limit of detection for the assay; in these cases, the result was set to the maximum readout value of the assay, 3.51.

Approximately 5% of the samples (*n* = 94) were randomly selected as laboratory-blinded replicates for quality control; the coefficient of variation was observed to be 5.0% (95% CI: 4.3–5.8%) and the intraclass correlation coefficient was 0.97 (95% CI: 0.96–0.98).

### Laboratory methods for HPV DNA testing

HPV DNA detection and testing were performed at DDL Diagnostic Laboratory (Delft, Netherlands) using cervical specimens collected at the time of vaccination. The presence of HPV DNA was detected by a polymerase chain reaction amplification with SPF10 primer sets. Using the same SPF10 primer sets, HPV genotyping of 25 HPV types, including HPV16 and 18, was conducted using reverse hybridization on a line probe assay (LiPA, Labo Bio-medical Products, Rijswijk, Netherlands) among SFP10-DNA-enzyme-immunoassay-positive samples.

### Statistical analysis

Our study aimed to address three research questions: (1) Does avidity change over the course of 11 years? (2) How does antibody avidity compare between one-dose and three-dose participants? (3) Does the avidity response differ between participants who were seronegative and those who were seropositive at first HPV vaccination (i.e., exposed to HPV prior to vaccination)? With the available data, we also evaluated if the relationship between antibody titer and avidity changed over the course of 11 years. Lastly, we examined vaccine efficacy against incident, 6-month persistent HPV16 infections among HPV16 DNA-negative women who were HPV16 seronegative versus HPV16 seropositive at the time of vaccination over the first four years of follow-up among one-dose and three-dose participants.

The main analysis focused on women who were HPV16 seronegative at first HPV vaccination. There were a small number of women (*N* = 5) who were HPV-seronegative but HPV DNA-positive at first HPV vaccination. We included them in our analysis, as most of these cases were likely transient HPV infections or deposition and would not affect our study on avidity.

We first described the antibody avidity over time. We reported the avidity of anti-HPV16-VLP antibodies for one-dose and three-dose women by calculating the GMA at each study visit (Years 0, 1, 2, 3, 4, 7, 9, and 11). We also reported the percent change in GMA between two consecutive visits and summarized results for the later years (Years 4–11) by the average percent change per year. We note that all estimates and their CI came from regressing log(avidity) on either an intercept, an indicator for the second study visit, and/or study year, and reporting on the parameter 100(e^β^−1). Generalized Estimating Equations (GEEs) were used in all analyses to account for within-subject correlation. We next compared the avidity between the one-dose and three-dose groups; we reported the ratio between GMAs at each study visit and averaged overall visits. We note these estimates came from regressing log(avidity) on dose group, adjusting for study year as a categorical variable when obtaining the average estimate, and reporting on e^β^. We then compared the avidity response of HPV-seronegative women to HPV-seropositive (at first HPV vaccination) women within each dose group; we reported the ratio between GMAs using similar methods.

One-dose and three-dose vaccine efficacy was calculated through 4 years of follow-up after initial vaccination. The analytical cohort included all women who received either one or three doses of the HPV16/18 vaccine and were HPV16 DNA negative from time of vaccination through 2-year post-vaccination, stratified by HPV16 serological status (seropositive versus seronegative) at the time of vaccination. The endpoint was incident, 6-month persistent HPV16 infections, defined as the detection of HPV16 DNA in consecutive cervical samples collected over any 6-month period with no intervening negatives, detected at year 2 or later post-vaccination. Event counting started at year 2 to avoid misclassifying undetected prevalent infections at the time of vaccination as breakthrough infections.

For sensitivity analysis, we first repeated the above analyses restricting the percent change results to women with results at both visits being compared. Second, we compared antibody avidity with HPV16 serum antibody levels generated from previous ELISA testing of these samples^3–6^ by evaluating the Pearson correlation coefficient between the log-antibody avidity and log-antibody level among women who were seronegative at first HPV vaccination. To obtain an average correlation over all study years, we normalized all measurements to have a mean = 0 and variance = 1, regressed log-avidity on log-antibody levels using a GEE and reported on β. Third, we evaluated the variability of avidity levels within an individual using “spaghetti” plots and analyses described in the supplementary material. The statistical package used for our analyses is SAS 9.4.

### Reporting summary

Further information on research design is available in the Nature Research Reporting Summary linked to th﻿is article.

## Supplementary information

NPJVACCINES-01464R_Supplemental_Avidity 082521 RESUBMIT_Clean

REPORTING SUMMARY

## Data Availability

Participant data can be shared with outside collaborators for research to understand more about the performance of the HPV vaccine, immune response to the vaccine, and broader study factors associated with the natural history of HPV infection and risk factors for infection and disease. Outside collaborators can apply to access our protocols and data from the blinded phase of the Costa Rica Vaccine Trial (NCT00128661). Outside collaborators can apply for access to the data online. Data for the long-term follow-up phase are not yet available. For the trial summary, current publications, and contact information for data access see: Human Papillomavirus (HPV) Vaccine Trial in Costa Rica (CVT) - National Cancer Institute. The data that support the findings of this study are available from the corresponding author upon reasonable request.
