# Supplementary material for: HPV16 infection decreases vaccine-induced HPV16 antibody avidity: the CVT trial
Source: NPJ Vaccines. 2022 Mar 29;7:40. doi: 10.1038/s41541-022-00431-x (PMC8964739; doi:10.1038/s41541-022-00431-x)
Supplement: Supplementary file 1 — NPJVACCINES-01464R_Supplemental_Avidity 082521 RESUBMIT_Clean [file 41541_2022_431_MOESM1_ESM.pdf]

HPV16 Infection Decreases Vaccine-Induced HPV16 Antibodies: The CVT trial

Tsang et al.

Supplementary information

Supplementary Table 1. Characteristics of 1-dose and 3-dose participants in the subset selected for avidity tested and compared to the full cohort.

Characteristics	1-dose				3-dose			
	Selected		All		Selected		All	
	N	%	N	%	N	%	N	%
Age at enrollment								
18-21	112	56.6	163	59.3	181	56.4	1679	56.6
22-25	86	43.4	112	40.7	140	43.6	1285	43.4
HPV16 serostatus at first HPV vaccination								
Positive	57	28.8	72	26.7	80	24.9	741	25.3
Negative	141	71.2	198	73.3	241	75.1	2185	74.7
HPV18 serostatus at first HPV vaccination								
Positive	51	26.3	66	24.6	76	24.3	694	23.9
Negative	143	73.7	202	75.4	237	75.7	2205	76.1
HPV DNA status at first HPV vaccination								
HPV16/18 positive	26	13.2	31	11.3	16	5.0	223	7.5
Any HPV positive	70	35.5	93	33.9	97	30.5	916	31.0
HPV negative	101	51.3	150	54.7	205	64.5	1819	61.5
Number of follow-up visits attended (not including clinical mgmt. visits)								
0-1	0	0.0	57	20.7	0	0.0	111	3.7
2-3	44	22.2	55	20.0	8	2.5	265	8.9
4-5	54	27.3	61	22.2	54	16.8	680	22.9
6-7	100	50.5	102	37.1	259	80.7	1908	64.4
Number of follow-up visits that yielded a sample for avidity testing								
0-1	0	0.0			0	0.0		
2-3	91	46.0			184	57.3		
4-5	76	38.4			90	28.0		
6-7	31	15.7			47	14.6		
Body mass index, BMI								
Underweight (<18.5 kg/m^2)	11	5.6	18	6.5	25	7.8	215	7.3
Normal weight (18.5-24.9 kg/m^2)	109	55.1	164	59.6	171	53.3	1681	56.7
Overweight (25-29.9 kg/m^2)	52	26.3	65	23.6	66	20.6	648	21.9
Obese (>= 30 kg/m^2)	26	13.1	28	10.2	59	18.4	420	14.2

Supplementary methods. Change in Avidity Over Time: Individual-Level Heterogeneity

In our cohort, the Geometric Mean Avidity (GMA) remained relatively constant between years 2 and 11. However, this could result from (i) avidity levels in all participants remaining relatively constant over time or (ii) avidity levels increasing in some participants but decreasing in other participants (i.e. heterogeneity). As described next, we found little evidence to support the presence of significant heterogeneity.

We first show the "spaghetti plots" of avidity levels (Supplementary Figure 3). Each line in the plot shows the avidity levels for a given participant over time. We note that most lines are relatively flat. We next calculated participant level slopes ($\hat{\beta}_i$) for individuals with at least 4 measurements spanning at least 5 years. We obtained the participant's slopes by regressing a participant's log-avidity levels on study year using data from years 3 to 11. As the spaghetti plots indicated, most slopes were close to 0 (Supplementary Figures 4, 5). Finally, we allowed for subject-specific random effects of the time coefficient in our regression models. We fit the mixed model: $\log(Y_{ijk}) = \gamma_0 + \gamma_t T_{ij} + \gamma_{0i} + \gamma_{ti} T_{ij} + \epsilon_{ij} + e_{ijk}$, where *i* indexes participant, *j* indexes measurement, *k* indexes replicate (i.e. some samples were run twice), T_{ij} is the study year, Y_{ijk} is the avidity level, and the random effects are $\gamma_{0i} \sim N(0, \sigma_0^2)$, $\gamma_{ti} \sim N(0, \sigma_t^2)$, $\epsilon_{ij} \sim N(0, \sigma_\epsilon^2)$, and $e_{ijk} \sim N(0, \sigma_e^2)$. Again, we found that σ_t was small compared to σ_ϵ or σ_0 (see Supplementary Table 4), suggesting that the effect of time (i.e. $\gamma_t + \gamma_{ti}$) varied little across participants. Coupled with $\gamma_t \approx 0$, avidity levels were relatively constant over time for all subjects.

(a) 1-dose

1-dose & Seronegative (Group 1)

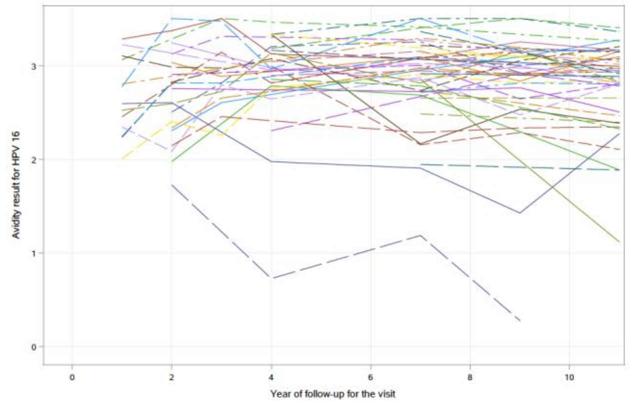

1-dose & Seronegative (Group 2)

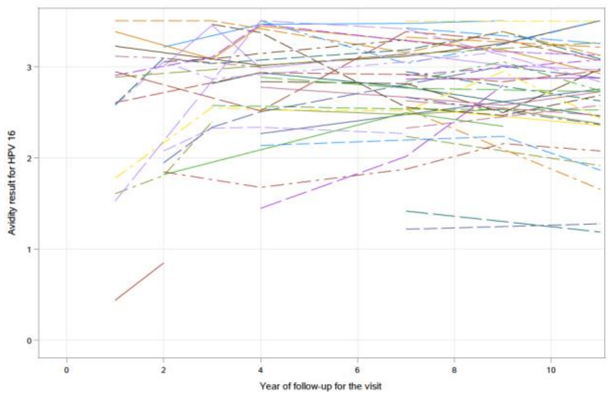

1-dose & Seronegative (Group 3)

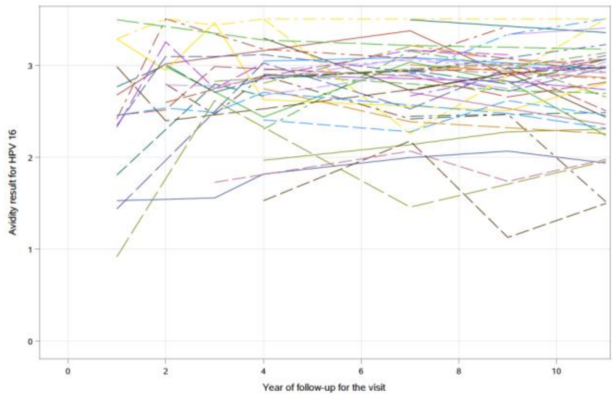

1-dose & Seropositive

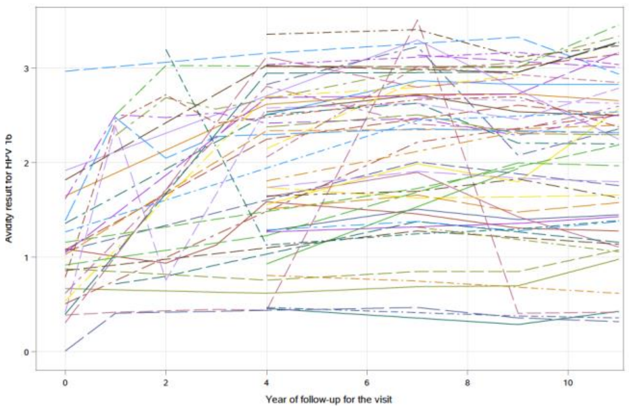

(b) 3-dose

3-dose & Seronegative (Group 1)

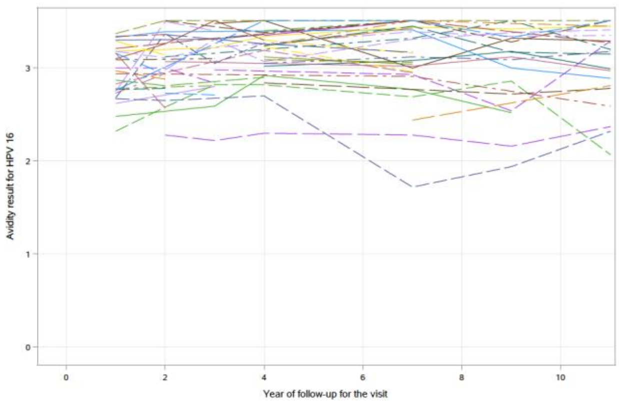

3-dose & Seronegative (Group 2)

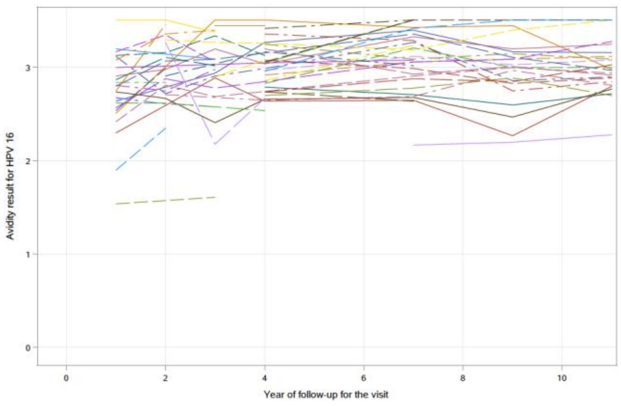

3-dose & Seronegative (Group 3)

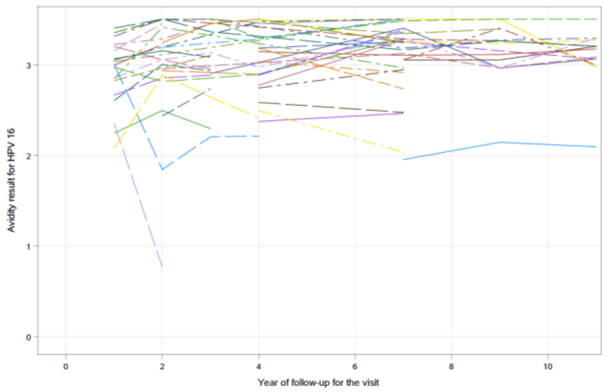

3-dose & Seronegative (Group 4)

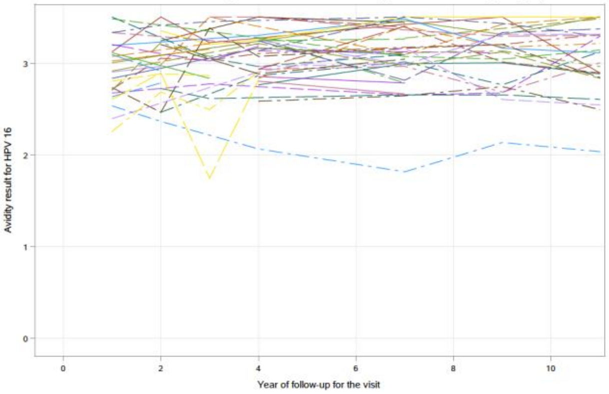

3-dose & Seronegative (Group 5)

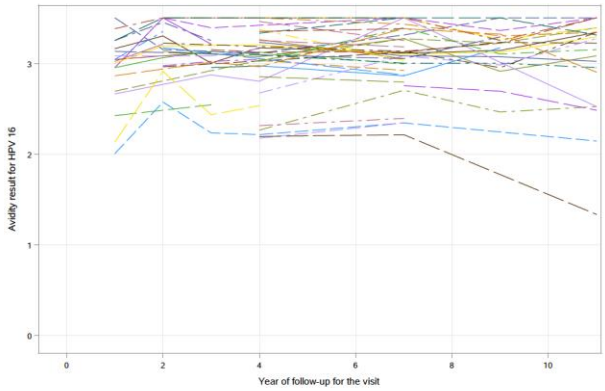

3-dose & Seropositive (Group 1)

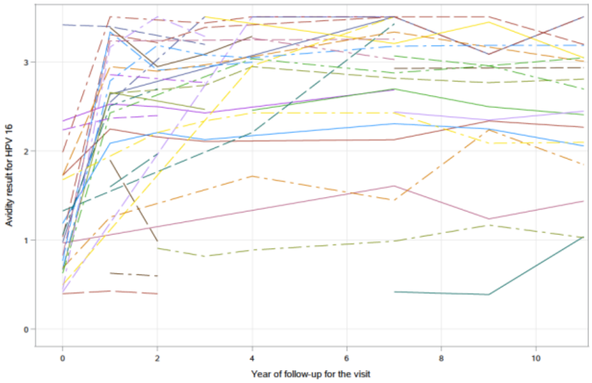

3-dose & Seropositive (Group 2)

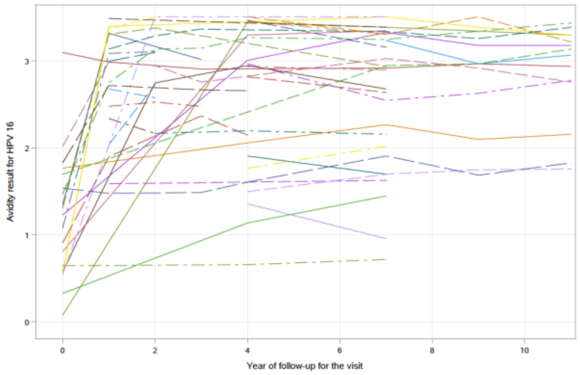

Supplementary Figure 1. Spaghetti Plots. Each line shows one participant's avidity levels over time by vaccine dose [(a) 1-dose and (b) 3-dose]. Subjects are categorized by number of vaccine doses received and baseline HPV-16 serology. Individuals in each category were randomly split into multiple groups for ease of display.

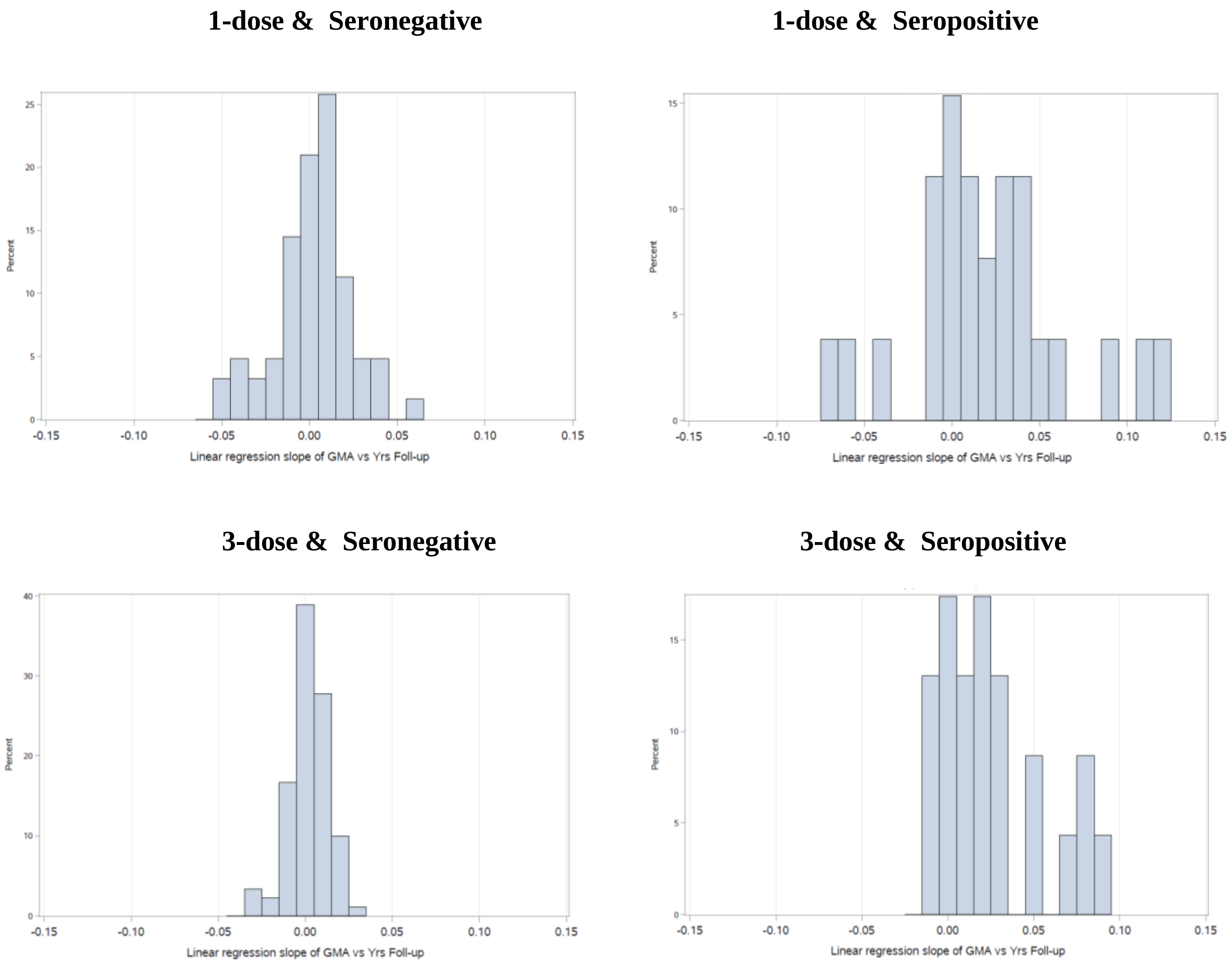

Supplementary Figure 2. Histogram of participant level slopes. The figures show the distribution of slopes between $\hat{\beta}_i$= -0.05 and $\hat{\beta}_i$= 0.05. Subjects are categorized by number of vaccines received and baseline HPV-16 serology. For outliers, please see supplementary figure 5.

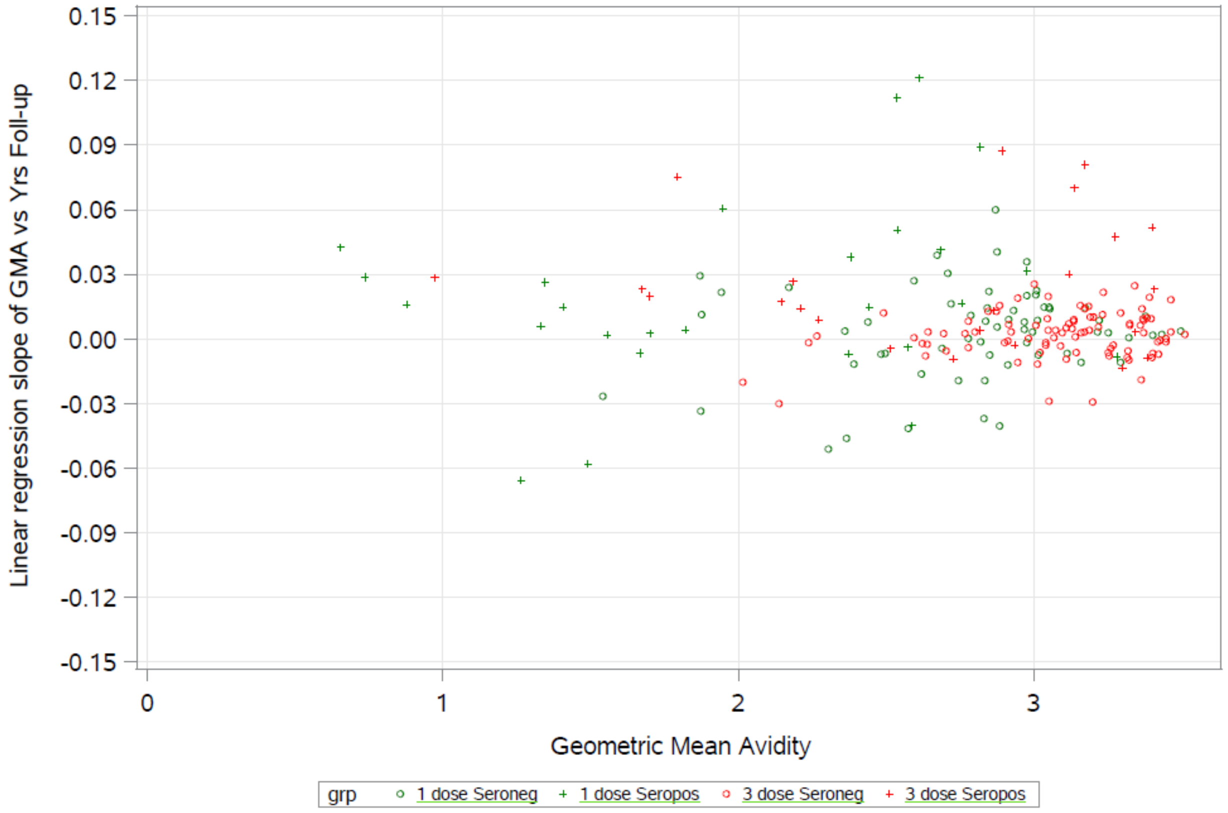

Supplementary Figure 3. Avidity levels. Plot of participants' mean avidity level (x-axis) vs participants' slopes (y-axis). The color indicates the number of HPV vaccine doses and the symbol indicates the baseline HPV-16 serology status.

Supplementary Table 2. Parameter estimates from linear mixed models.

# DOSES	Baseline Serology	γ_0	γ_t	σ_0^2	σ_t^2	σ_ϵ^2
1	+	0.56 (0.078)	0.0066 (0.0056)	0.23	0	0.041
1	-	1.04 (0.021)	-0.0043 (0.0023)	0.023	0.00015	0.011
3	+	0.92 (0.059)	0.0033 (0.0027)	0.21	0.000036	0.0084
3	-	1.10 (0.010)	0.00020 (0.0012)	0.012	0.000042	0.00096

The first two columns define the cohort, while the remaining columns show the estimated parameters.

Standard errors for fixed effects are provided in parentheses.

Supplementary Table 3. Correlation between antibody level and avidity among 1-dose and 3-dose participants who were HPV16 seronegative or seropositive at first HPV vaccination.

	1-dose		3-dose	
	Seronegative	Seropositive †	Seronegative ‡	Seropositive ‡‡
Year of followup	PCC (95% CI)	PCC (95% CI)	PCC (95% CI)	PCC (95% CI)
First HPV vaccination	N/A	-0.15 (-0.87- 0.58)	N/A	0.33 (-0.08- 0.73)
Year 1	0.27 (-0.04- 0.59)	-0.47 (-1.07- 0.13)	0.20 (0.04- 0.35)	0.26 (0.10- 0.42)
Year 2	0.15 (-0.14- 0.45)	0.13 (-0.38- 0.64)	0.11 (-0.10- 0.33)	0.51 (0.04- 0.97)
Year 3	0.02 (-0.27- 0.30)	-0.28 (-0.96- 0.41)	0.14 (-0.03- 0.30)	0.27 (-0.18- 0.73)
Year 4	-0.06 (-0.24- 0.12)	-0.08 (-0.50- 0.33)	0.28 (0.10- 0.45)	0.00 (-0.35- 0.35)
Year 7	0.13 (-0.04- 0.30)	-0.06 (-0.33- 0.20)	0.22 (0.06- 0.38)	0.05 (-0.17- 0.27)
Year 9	-0.10 (-0.27- 0.07)	-0.01 (-0.34- 0.31)	0.23 (0.04- 0.43)	0.14 (-0.13- 0.40)
Year 11	-0.13 (-0.38- 0.12)	0.05 (-0.20- 0.31)	0.37 (0.07- 0.68)	0.30 (-0.05- 0.65)
**Overall**	**0.04 (-0.10- 0.18)**	**0.08 (-0.09- 0.26)**	**0.14 (0.01- 0.27)**	**0.44 (0.31- 0.57)**

PCC, Pearson correlation coefficient; CI, confidence intervals; N/A, not applicable

† 19 ELISA results missing

‡ 11 ELISA results missing from Year 2

‡‡ 22 ELISA results missing from first HPV vaccination, 9 ELISA results missing from Year 2

a) 1-dose women who were seronegative at first HPV vaccination

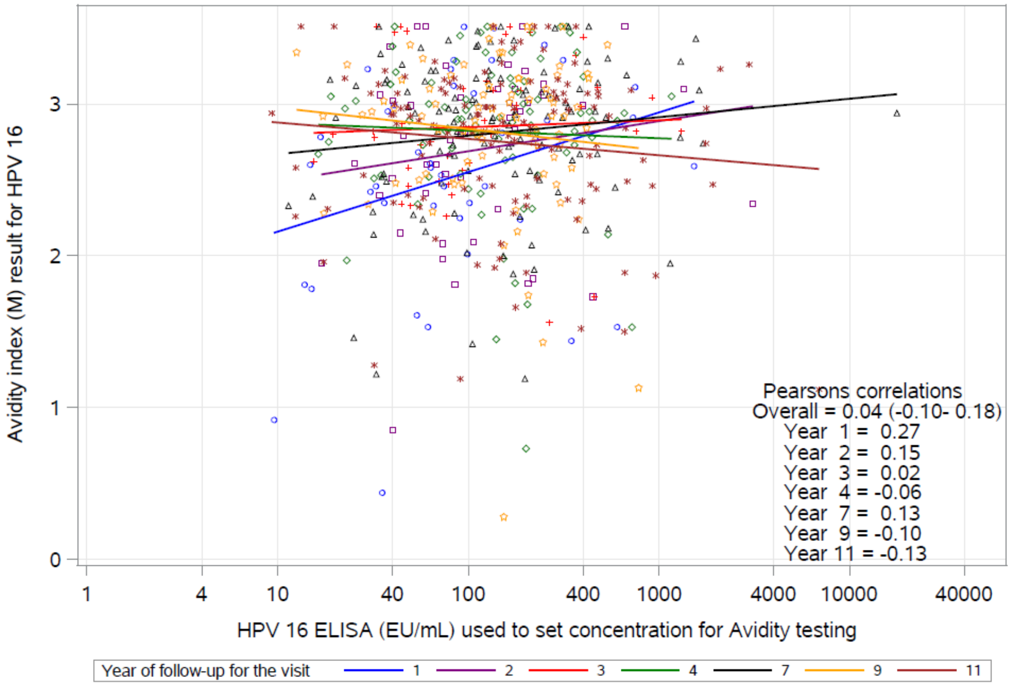

b) 3-dose women who were seronegative at first HPV vaccination

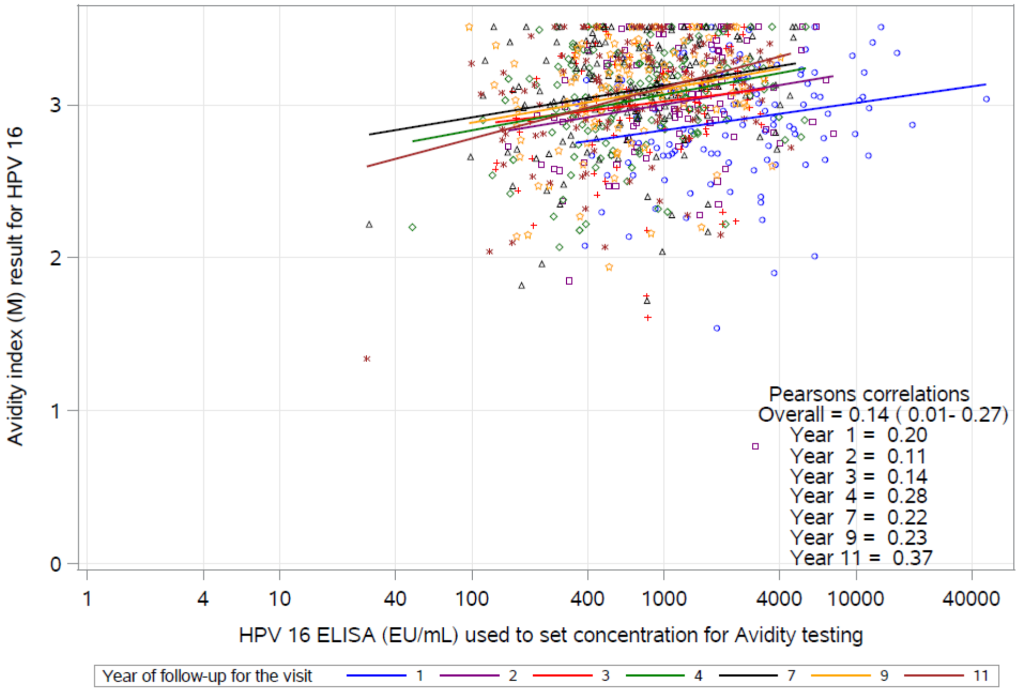

Supplementary Figure 4. Correlation between antibody level and avidity by vaccine dose. Antibody levels measured by ELISA. **a** 1-dose women who were seronegative at first HPV vaccination. **b** 3-dose women who were seronegative at first HPV vaccination.

a) 1-dose women who were seropositive at first HPV vaccination

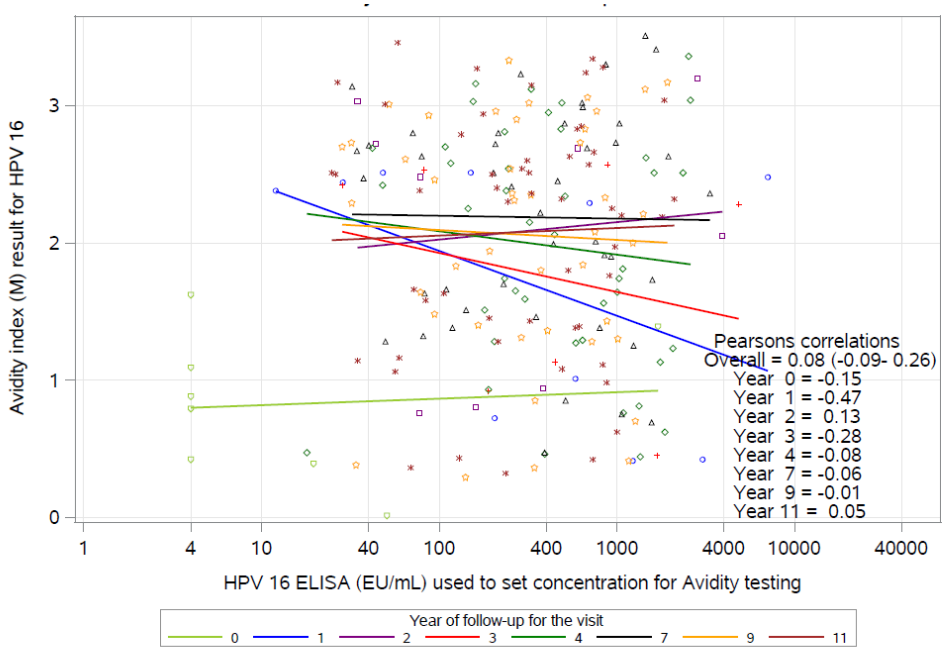

b) 3-dose women who were seropositive at first HPV vaccination

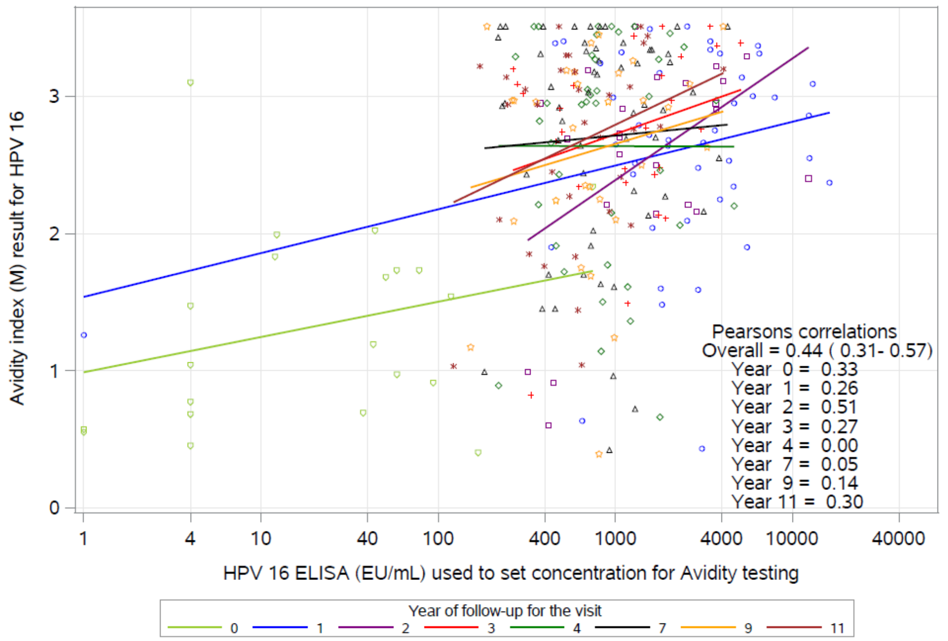

Supplementary Figure 5. Correlation between antibody level (measured by ELISA) and avidity by vaccine dose. Antibody levels measured by ELISA. **a** 1-dose women who were seropositive at first HPV vaccination. **b** 3-dose women who were seropositive at first HPV vaccination.
