# Supplementary material for: HPV16 infection decreases vaccine-induced HPV16 antibody avidity: the CVT trial
Source: NPJ Vaccines. 2022 Mar 29;7:40. doi: 10.1038/s41541-022-00431-x (PMC8964739; doi:10.1038/s41541-022-00431-x)
Supplement: Supplementary file 2 — REPORTING SUMMARY [file 41541_2022_431_MOESM2_ESM.pdf]

nature portfolio

Corresponding author(s): DBPR NPJVACCINES-01464R

Last updated by author(s): Aug 25, 2021

Reporting Summary

Nature Portfolio wishes to improve the reproducibility of the work that we publish. This form provides structure for consistency and transparency in reporting. For further information on Nature Portfolio policies, see our Editorial Policies and the Editorial Policy Checklist.

Statistics

For all statistical analyses, confirm that the following items are present in the figure legend, table legend, main text, or Methods section.

n/a	Confirmed	
☐	☒	The exact sample size (*n*) for each experimental group/condition, given as a discrete number and unit of measurement
☐	☐	A statement on whether measurements were taken from distinct samples or whether the same sample was measured repeatedly
☐	☒	The statistical test(s) used AND whether they are one- or two-sided<br>*Only common tests should be described solely by name; describe more complex techniques in the Methods section.*
☒	☐	A description of all covariates tested
☐	☒	A description of any assumptions or corrections, such as tests of normality and adjustment for multiple comparisons
☐	☒	A full description of the statistical parameters including central tendency (e.g. means) or other basic estimates (e.g. regression coefficient) AND variation (e.g. standard deviation) or associated estimates of uncertainty (e.g. confidence intervals)
☐	☒	For null hypothesis testing, the test statistic (e.g. F, t, r) with confidence intervals, effect sizes, degrees of freedom and P value noted<br>*Give P values as exact values whenever suitable.*
☒	☐	For Bayesian analysis, information on the choice of priors and Markov chain Monte Carlo settings
☒	☐	For hierarchical and complex designs, identification of the appropriate level for tests and full reporting of outcomes
☐	☒	Estimates of effect sizes (e.g. Cohen's *d*, Pearson's *r*), indicating how they were calculated

Our web collection on statistics for biologists contains articles on many of the points above.

Software and code

Policy information about availability of computer code

Data collection	Data capture was paper based at that time. Custom built software was used to manage the field efforts for both the trial and observational portions of the study. For this analysis
Data analysis	SAS 9.4

For manuscripts utilizing custom algorithms or software that are central to the research but not yet described in published literature, software must be made available to editors and reviewers. We strongly encourage code deposition in a community repository (e.g. GitHub). See the Nature Portfolio guidelines for submitting code & software for further information.

Data

Policy information about availability of data

All manuscripts must include a data availability statement. This statement should provide the following information, where applicable:

- Accession codes, unique identifiers, or web links for publicly available datasets
- A description of any restrictions on data availability
- For clinical datasets or third party data, please ensure that the statement adheres to our policy

Participant data can be shared with outside collaborators for research to understand more about the performance of the HPV vaccine, immune response to the vaccine, and broader study factors associated with the natural history of HPV infection and risk factors for infection and disease. Outside collaborators can apply to access our protocols and data from the blinded phase of the Costa Rica Vaccine Trial (NCT00128661). Outside collaborators can apply for access to the data online. Data for the long-term follow-up phase are not yet available. For the trial summary, current publications, and contact information for data access see: Human Papillomavirus (HPV) Vaccine Trial in Costa Rica (CVT) - National Cancer Institute.
The data that support the findings of this study are available from the corresponding author upon reasonable request.

Field-specific reporting

Please select the one below that is the best fit for your research. If you are not sure, read the appropriate sections before making your selection.

☒ Life sciences ☐ Behavioural & social sciences ☐ Ecological, evolutionary & environmental sciences

For a reference copy of the document with all sections, see nature.com/documents/nr-reporting-summary-flat.pdf

Life sciences study design

All studies must disclose on these points even when the disclosure is negative.

Sample size	98 women (18-25 years) who received 1-dose of HPV vaccine (with 747 follow-up samples) and 321 women (18-25 years) who received 3-dose of the HPV vaccine (with 1,115 follow-up samples).
Data exclusions	For this ancillary study, we focused on the enrollment and follow up study visits at Years 0, 1, 2, 3, 4, 7, 9, and 11. All 8 timepoints were included to fully describe the long-term kinetics of antibody avidity over time. Avidity at Year 0 (pre-vaccination; enrollment visit) was only evaluated in women who were HPV16 seropositive at first HPV vaccination, providing insight into the avidity of natural infection-induced antibody and the effect of prior infection on avidity responses to the vaccine. Our selection was based on availability of prior IgG ELISA results for HPV16 (tested over the course of multiple CVT studies3,4,16,23-26 at the Frederick National Laboratory for Cancer Research, Frederick, Maryland), necessary to control for the concentration of antibody added to the avidity assay, and further restricted to those who contributed two or more serum samples over the course of 11 years (excluding the enrollment visit). After applying these inclusion criteria, our selection yielded 198 1-dose women (with 747 follow-up samples) and 321 3-dose women (with 1,115 follow-up samples).
Replication	Approximately 5% of the samples (n = 94) were randomly selected as laboratory-blinded replicates for quality control; the coefficient of variation (CV) was observed to be 5.0% (95% CI: 4.3-5.8%) and the intraclass correlation coefficient (ICC) was 0.97 (95% CI: 0.96-0.98).
Randomization	This is an ancillary study. But women enrolled in the trail in 2004-2005 were randomized in 1:1 ratio to receive either the AS04-adjuvanted HPV16/18 vaccine (CervarixTM; GlaxoSmithKline Biologicals, Rixensart, Belgium) or a control hepatitis A vaccine (GlaxoSmithKline Biologicals)
Blinding	The original study is a randomized blinded control trial. For this ancillary study, laboratory personnel conducting the avidity experiments were blinded to vaccination status and blinded replicated samples.

Reporting for specific materials, systems and methods

We require information from authors about some types of materials, experimental systems and methods used in many studies. Here, indicate whether each material, system or method listed is relevant to your study. If you are not sure if a list item applies to your research, read the appropriate section before selecting a response.

Materials & experimental systems

n/a	Involved in the study	
☒	☐	Antibodies
☒	☐	Eukaryotic cell lines
☒	☐	Palaeontology and archaeology
☒	☐	Animals and other organisms
☐	☒	Human research participants
☐	☒	Clinical data
☒	☐	Dual use research of concern

Methods

n/a	Involved in the study	
☒	☐	ChIP-seq
☒	☐	Flow cytometry
☒	☐	MRI-based neuroimaging

Human research participants

Policy information about studies involving human research participants

Population characteristics	Women 18-25 years of age living in Costa Rica between 2004 and 2005.
Recruitment	Details of recruitment for the Costa Rica HPV Vaccine Trial and the long-term follow-up are reported in Ref#21 and Ref#23. This is an ancillary study to test antibody avidity levels of historically collected samples.
Ethics oversight	U.S. National Cancer Institute (NCI) Institutional Review Boards and the corresponding Costa Rican Institutional Review Board.

Note that full information on the approval of the study protocol must also be provided in the manuscript.

Clinical data

Policy information about clinical studies

All manuscripts should comply with the ICMJE guidelines for publication of clinical research and a completed CONSORT checklist must be included with all submissions.

Clinical trial registration	NCT00128661
Study protocol	Study protocol can be accessed upon request.
Data collection	Between 2004 and 2005, 7,466 women 18-25 years of age in Costa Rica were enrolled and randomized to receive either the AS04-adjuvanted HPV16/18 vaccine (CervarixTM; GlaxoSmithKline Biologicals, Rixensart, Belgium) or a control hepatitis A vaccine (GlaxoSmithKline Biologicals) in a 1:1 ratio at 0, 1, and 6 months, and were followed for four years in the Costa Rica HPV Vaccine Trial (CVT). At enrollment and annual follow-up visits, participants provided a serum sample, and for sexually experienced women, a pelvic exam was performed at which cervical cells were collected for cytology and HPV DNA testing.<br>At the end of the blinded phase, participants in the HPV-vaccinated arm were invited to stay in the CVT observational study22 and followed biennially in years 7, 9, and 11, when each clinic visit consisted of a pelvic exam with collection of a cervical sample and a serum sample, for virologic and immunologic endpoint assessments, respectively.
Outcomes	For this ancillary study, the outcomes are: Geometric Mean of the Avidity Index over the course of 11 years; 2) HPV16 antibody avidity over time, stratified by number of HPV vaccine doses received and HPV16 serostatus at time of initial HPV vaccination; and 3) HPV16 antibody avidity over time among HPV16 seronegative at initial HPV vaccination, stratified by number of HPV vaccine doses received and HPV18 serostatus at time of initial HPV vaccination
